# Dissipative Particle Dynamics Simulation of Ultrasound Propagation through Liquid Water

**DOI:** 10.1021/acs.jctc.1c01020

**Published:** 2022-01-10

**Authors:** Petra Papež, Matej Praprotnik

**Affiliations:** † Laboratory for Molecular Modeling, National Institute of Chemistry, Hajdrihova 19, Ljubljana, SI-1001, Slovenia; ‡ Department of Physics, Faculty of Mathematics and Physics, University of Ljubljana, Jadranska 19, Ljubljana, SI-1000, Slovenia

## Abstract

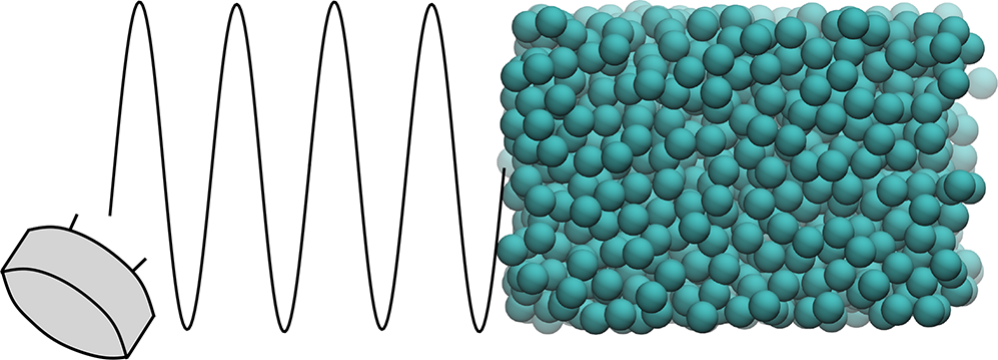

Ultrasound is widely used as a noninvasive method in therapeutic and diagnostic applications. These can be further optimized by computational approaches, as they allow for controlled testing and rational optimization of the ultrasound parameters, such as frequency and amplitude. Usually, continuum numerical methods are used to simulate ultrasound propagating through different tissue types. In contrast, ultrasound simulations using particle description are less common, as the implementation is challenging. In this work, a dissipative particle dynamics model is used to perform ultrasound simulations in liquid water. The effects of frequency and thermostat parameters are studied and discussed. We show that frequency and thermostat parameters affect not only the attenuation but also the computed speed of sound. The present study paves the way for development and optimization of a virtual ultrasound machine for large-scale biomolecular simulations.

## 1 Introduction

Ultrasound consists of mechanical pressure waves, which can propagate through various media with frequencies above the upper limit of (average) human hearing, that is, above 20 kHz.^1−4^ Unlike light, which is scattered roughly within 1 mm of tissue, ultrasound easily penetrates centimeters deep while maintaining spatial and temporal coherence.^2,5^ For this reason, ultrasound is used in many medical applications. Nonetheless, ultrasound is also used in, for example, nanotechnology, sonochemistry,^6,7^ food processing, industrial processes (e.g., welding), and nondestructive material investigation.^8,9^ In medical applications, it is commonly used as a safe and noninvasive diagnostic (imaging) tool to diagnose many types of cancers, such as breast, stomach, and thyroid. Additionally, it is employed in therapeutic applications in cases of joint inflammation, rheumatoid arthritis, mechanical tissue disruption, kidney stone comminution, bone healing, and as an alternative treatment to the surgical resection of tumors.^10−15^

Due to its applicability, there is also a need for simulation methods that provide an insight into the phenomena that occur during ultrasound treatment or tissue imaging, and thus open the door to clinical applications. In most methods the assumption of isotropic, nondissipative, and homogeneous medium is made. However, these assumptions are typically oversimplistic and the computational cost is too high.^16−19^ To this end, computationally more efficient methods incorporating heterogeneous tissue properties have also been proposed.^18,20−26^

Moving away from diagnostic ultrasound simulations, sound waves are simulated using classical mesh-based numerical methods, for example, the boundary element method (BEM),^27^ the finite element method (FEM),^28,29^ and their modifications.^30,31^ On the other hand, also meshless methods are used, for example, the method of fundamental solutions (MFS),^32^ the multiple-scale reproducing kernel particle method (RKPM),^33^ and the element free Galerkin method (EFG).^34^ In addition, the smoothed particle hydrodynamics method (SPH) has proven to be a promising particle-based method for sound simulations. It is able to accurately model the sound propagation, and the effects of sound frequency, maximum sound pressure amplitude, and particle spacing on the numerical error and time consumption were studied by Zhang et al.^35,36^ Another contribution to simulations of ultrasound waves using a particle-based description^37^ was made by De Fabritiis et al.^38,39^ They proposed a coupled multiscale model, that is, hybrid molecular dynamics (MD). In the hybrid MD, the mesoscopic description of a fluid flow, based on the equations of fluctuating hydrodynamics (FH), is coupled with the molecular description of particles. By successfully coupling FH and classical MD, they have overcome the limitations of already existing hybrid descriptions of liquids that were limited to the coarse-grained (CG) descriptions based on the Lennard-Jones particles. Using the proposed hybrid MD method, they simulated sound waves, generated as the Gaussian density perturbation of the equilibrium state, in bulk water reflected by a lipid monolayer. Furthermore, Korotkin et al.^40^ suggested a new method, that is, a hybrid MD/FH method, based on the two-phase flow analogy. The method smoothly combines the atomistic (AT) description in the MD zone with the Landau-Lifshitz fluctuating hydrodynamics (LL-FH) representation in the rest of the system. The simulation domain is divided into cells in all three directions, where the pure MD zone in the center of the simulation domain is surrounded by two Landau-Lifshitz domains. The boundary condition of the sound wave is introduced by adding the analytical source terms to the governing LL-FH equations for cells at the beginning of the simulation domain. The analytical source terms correspond to the time derivatives of the density and velocity of the incoming sound wave of small amplitude propagating over the prescribed constant mean flow field of the LL-FH solution. In a consecutive work, Hu et al.^41^ extended the already existing hybrid MD/FH method^40^ with the scale-bridging adaptive resolution scheme (AdResS). Additionally, Korotkin and Karabasov^42^ developed the generalized LL-FH (GLL-FH) as the extension of the classical continuum LL-FH model in statistical mechanics. In the GLL-FH equations, compared to the classical LL-FH method, some additional time dependent solution variables are introduced, which describe the difference between the locally averaged fields, obtained by the MD, and the solution of continuum hydrodynamics.

In this work, we employ the mesoscopic dissipative particle dynamics (DPD) water model to perform the particle-based ultrasound simulations in the THz frequency range. To the best of our knowledge, there is no study available to examine the effects of ultrasound frequency, amplitude, and thermostat parameters on the propagation of ultrasound waves using the DPD model. Furthermore, ultrasound waves can be in general considered either as adiabatic or isothermal. In gases, low frequency sound waves are typically adiabatic, while the high frequency ones are isothermal.^43,44^ However, in water, it is not a priori clear which classification is more appropriate for ultrasound propagation in the THz frequency range. We aim to clarify this issue by conducting our simulations. Finally, we will also test the propagation of ultrasound waves through water, described by the simple point charge (SPC) model,^45^ using AdResS.

## 2 Theoretical Background and Methodology

### 2.1 Ultrasound

The dynamics of a viscous fluid is governed by the Navier−Stokes equation:
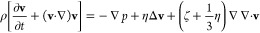
1where ρ stands for the fluid density, *p* is for pressure, *t* is for time, and **v** represents the fluid velocity. Coefficients ζ and η are positive, and represent the second and dynamic viscosity, respectively. For the incompressible fluid flow, the last term in the Navier–Stokes equation is omitted.^46^ If we further neglect the energy-dissipating second term on the right side in the Navier–Stokes equation, assume small oscillations, and consider the continuity equation, we obtain the wave equation for the velocity potential ϕ
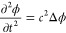
2where **v** = ∇ϕ. In the wave equation, *c* stands for the velocity of sound and is given by *c*^2^ = (*∂p*/*∂ρ*), either at constant entropy *s* or at constant temperature *T*. For instance, considering the monochromatic traveling plane wave, propagated in the positive direction of the *x*-axis, and introducing the wave vector **k** as **k** = (ω/*c*)**n** = (2π/λ)**n**, where **n** denotes a unit vector in the direction of the propagation of the sound wave and λ denotes the wavelength of the propagated sound wave, the solution of eq 2 is
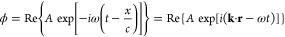
3where *A* = *ae*^*iφ*^ represents the complex amplitude, **r** is the position vector, and Re stands for the real part. Parameters ω, *a*, and φ are the frequency of the wave, amplitude, and phase shift, respectively.

The propagation of any sound wave through a medium is governed by the wave equation. In this work, we simulate ultrasound waves and compare the obtained signals with the solutions corresponding to eq 3. Ultrasound consists of mechanical pressure waves (*p* = −*ρ∂ϕ*/*∂t*), which can propagate through media with frequencies above the audible threshold of 20 kHz. From the experimental point of view, the most commonly varied ultrasound parameters are frequency, intensity, applied acoustic pressure, mechanical index (defined as the peak negative pressure divided by the square root of center frequency), and duration of the exposure to the ultrasound.^1,47−50^ Accordingly, the parameters of interest in our study are the frequency and amplitude of the oscillatory part of the simulated ultrasound waves.

As already mentioned, sound waves can be isothermal or adiabatic.^43,44^ Each medium has a frequency associated with thermal conduction (TC), expressed as ω_TC_ = *ρc*_*p*_*c*^2^/κ_TC_ = 2*πν*_TC_, where *c*_*p*_ and κ_TC_ stand for the heat capacity at constant pressure and coefficient of thermal conductivity, respectively. ω_TC_/2π for water is of the order of 2 THz.^44^ At very high frequencies, that is, if ω ≫ ω_TC_, the process of sound propagation can be considered as isothermal, while the adiabatic approximation is better at lower frequencies, that is, if ω ≪ ω_TC_.^43,44,51^ However, for water it is expected that there is a negligible difference between the isothermal or adiabatic approximation, as Δκ/κ ∼ 10^–4^. Besides, at THz frequencies corresponding wavelengths are comparable to the mean free path of water molecules and we approach the limit of validity of the thermodynamics.

### 2.2 Dissipative Particle Dynamics (DPD)

We simulate ultrasound on a mesoscopic level using a particle-based DPD method. It is particularly suitable for simulating liquids and soft matter since its linear momentum conserving equations of motion recover the Navier−Stokes equations in the continuum limit (FH).^52−54^ The CG nature of a DPD model enables simulations on larger time and length scales. The underlying idea of DPD is that many important properties of soft matter are determined by the collective properties of clusters of molecules rather by individual molecules.^55−57^ For instance, the DPD method has been applied to colloidal suspensions, multiphase flows, biological systems,^58−60^ and vesicle formation.^61^ The DPD equations of motion are
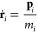
4and
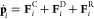
5**F**_*i*_^C^, **F**_*i*_^D^, and **F**_*i*_^R^ stand for the conservative, dissipative, and random force on the *i*th particle, respectively. They can be split into particle pair forces:
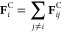
6
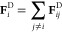
7and
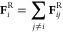
8The conservative force is
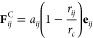
9where **r**_*ij*_ = **r**_*i*_ – **r**_*j*_, *r*_*ij*_ = |**r**_*ij*_|, **e**_*ij*_ = **r**_*ij*_/*r*_*ij*_, and **r**_*i*_, **r**_*j*_ are the position vectors of particle *i* and *j*, respectively. Parameter *a*_*ij*_ stands for the repulsion strength. This force becomes zero for *r*_*ij*_ ≥ *r*_c_, where *r*_c_ stands for the cutoff radius. The dissipative force is
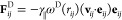
10and the random force is
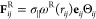
11where the relative velocity **v**_*ij*_ = **v**_*i*_ – **v**_*j*_ between two particles *i* and *j* is introduced. γ_∥_ is the friction constant and σ_∥_ is the noise strength. ω^R^(*r*_*ij*_), and ω^D^(*r*_*ij*_) are the *r*-dependent weight functions. They are related by the fluctuation–dissipation theorem:
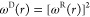
12
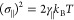
13and defined as
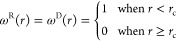
14Θ_*ij*_ in eq 11 is Gaussian white noise, symmetric in the particle indices (Θ_*ij*_ = Θ_*ji*_), with zero mean ⟨Θ_*ij*_(*t*)⟩ and unit variance ⟨Θ_*ij*_(*t*)Θ_*kl*_(*t*′)⟩ = (δ_*ik*_δ_*jl*_ + δ_*il*_δ_*jk*_)δ(*t* – *t*′), where ⟨.⟩ denotes the thermal average. In addition, the above DPD equations conserve the linear momentum and correctly reproduce the hydrodynamic interactions in the system.^62,63^ Together, dissipative and random forces act as a thermostat.^64,65^ We distinguish between two DPD thermostats, that is, the standard one (presented above) that acts on the relative velocities along the interatomic axis and the transverse dissipative particle dynamics (TDPD) thermostat that acts in perpendicular directions and enables viscosity tuning.^65^ In the TDPD thermostat, eq 10 is rewritten into
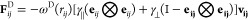
15and eq 11 is rewritten into
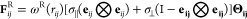
16where **Θ**_**ij**_ is the noise vector, defined as ⟨**Θ**_*ij*_(*t*) ⊗ **Θ**_*kl*_(*t*′)⟩ = I(δ_*ik*_δ_*jl*_ – δ_*il*_δ_*jk*_)δ(*t* – *t*′). The noise vector is antisymmetric in particles indices (**Θ**_*ij*_ = −**Θ**_*ji*_). Apart from relations in eqs 12 and 13, the relation (σ_⊥_)^2^ = 2γ_⊥_*k*_B_*T* needs to be fulfilled.^65^

### 2.3 Open Boundary Molecular Dynamics (OBMD)

To simulate ultrasound waves we need to open the molecular system so it can exchange mass, momentum, and energy with its surroundings. To achieve this goal, we resort to the OBMD.^66−70^ In OBMD, the simulation box is opened in one direction (or more), while the periodic boundary conditions are imposed in the remaining ones. The box is divided into three regions, where the central region, named the region of interest (ROI), is surrounded by two buffer regions. The latter act as particle reservoirs from which molecules are deleted and inserted into the system. The number of particles (or density) in the buffers is maintained by the feedback algorithm. The feedback algorithm is defined as Δ*N*_B_ = (*δt*/τ_B_)(⟨*N*_B_⟩ – *N*_B_), where ⟨*N*_B_⟩ represents the desired number of molecules inside the buffer and *N*_B_ stands for the current number of molecules inside the buffer. Parameter τ_B_ denotes the characteristic relaxation time of the buffers (). When Δ*N*_B_ < 0, molecules need to be deleted from the system. Conversely, when Δ*N*_B_ > 0, new molecules need to be inserted into the system. The insertion of new DPD particles is carried out by the iterative algorithm named USHER, which is a Newton-Raphson-like search method on the potential energy surface.^71,72^ The total linear momentum in the OBMD is conserved, which directly follows from the Navier−Stokes equation given by eq 1. The latter can be reformulated into a linear momentum conservation law as ∂(ρ **v**)/*∂t* = −∇·J^*P*^, where J^*P*^ stands for the momentum flux tensor, defined as J^*P*^ = ρ**u** ⊗ **u** + Π + Π̃. Here, Π and Π̃ are the mean and fluctuating contributions to the pressure tensor, respectively. The mean pressure is usually defined as Π = (*p* + π)I + Π^*S*^, where *p* stands for the pressure of the system (usually obtained from the equation of state), I represents the identity matrix, π represents the isotropic stress (π = −ζ∇·**u**), and Π^*S*^ is the traceless symmetric tensor, expressed as Π_*αβ*_^*S*^ = −η(∂_α_*u*_β_ + ∂_β_*u*_α_ – 2∂_γ_*u*_γ_δ_*αβ*_/*D*). Here, *D* represents the spatial dimension.^38,73^

OBMD imposes the external boundary conditions through buffers onto the ROI by an additional external force **f**_*i*_^ext^ that is applied only to the particles in the buffer regions; that is, **f**_*i*_^ext^ = 0 outside the buffer region. Boundary conditions are defined by the normal component of the energy flux, that is, the rate of energy transfer through a surface, as *J*_e_ = **J**_e_·**n**, where **J**_e_ represents the energy flux vector and **n** is the unit vector normal to the interface between buffer and ROI (pointing toward the center of ROI), and by the momentum flux J^*P*^·**n**, where J^*P*^ stands for the already defined momentum flux tensor. To determine external forces, the amount of momentum and energy created by these forces over one time step d*t* needs to be considered and the result equated to the desired amount of momentum and heat that needs to be added to or extracted from the system. The momentum balance of *A*, that is, the area of the interface between buffer and ROI, is
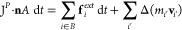
17and the energy balance is
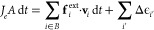
18In eqs 17 and 18, *i*′ runs over all particles that have been inserted into or deleted from the system in the last time step d*t*, while *i* runs over all particles that are within buffer regions. The momentum change Δ(*m*_*i*′_**v**_*i*′_) = *m*_*i*′_**v**_*i*′_ if the particle is inserted into the system and Δ(*m*_*i*′_**v**_*i*′_) = −*m*_*i*′_**v**_*i*′_ if the particle is deleted from the system. Similarily, this applies to the energy change Δϵ_*i*′_. The balance of eqs 17 and 18 ensures that the total momentum and energy are conserved. Boundary conditions impose the exact momentum and energy flux to the whole system (i.e., buffers + ROI). Since buffer has some mass and heat capacity, the momentum transfer across the interface between buffer and ROI is not instantaneously equal to the amount prescribed by the momentum and energy flux defined in eqs 17 and 18. However, in real applications this effect is usually negligible.^66,68^

To separate momentum from heat generation the external force **f**_*i*_^ext^ is divided into two parts:
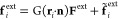
19where **F**^ext^ and **f̃**_*i*_^ext^ represent momentum and energy contributions, respectively. The force  is distributed among particles in the buffers, where G(**r**_*i*_·**n**) represents the distributing tensor: 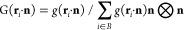
Note that **r**_*i*_·**n** is the distance from the interface in the *x*-direction. Using eq 17,
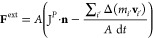
20The force **f̃**_*i*_^ext^ that gives no net momentum input, that is, , is
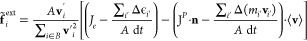
21where velocity **v**_*i*_′ = **v**_*i*_ – ⟨**v**⟩ and average velocity ⟨**v**⟩ = ∑_i∈B_**v***_i_*/*N*_*B*_.^66,74^

In this study, *J*_*e*_ is fixed or controlled via *J*_*e*_ = −λ(*T* – *T*_0_),^66^ where *T* stands for the current buffer temperature, *T*_0_ is its desired temperature, and λ represents the adjustable relaxation parameter. Note that the external boundary conditions are introduced into the system without modification of Newton’s equations of motion for particles in the bulk.^68,75^


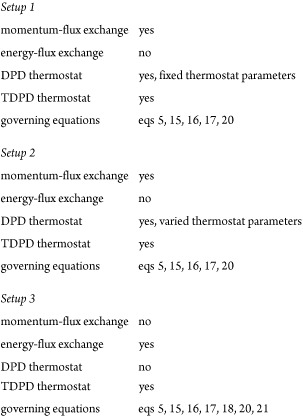


**Figure 1:**
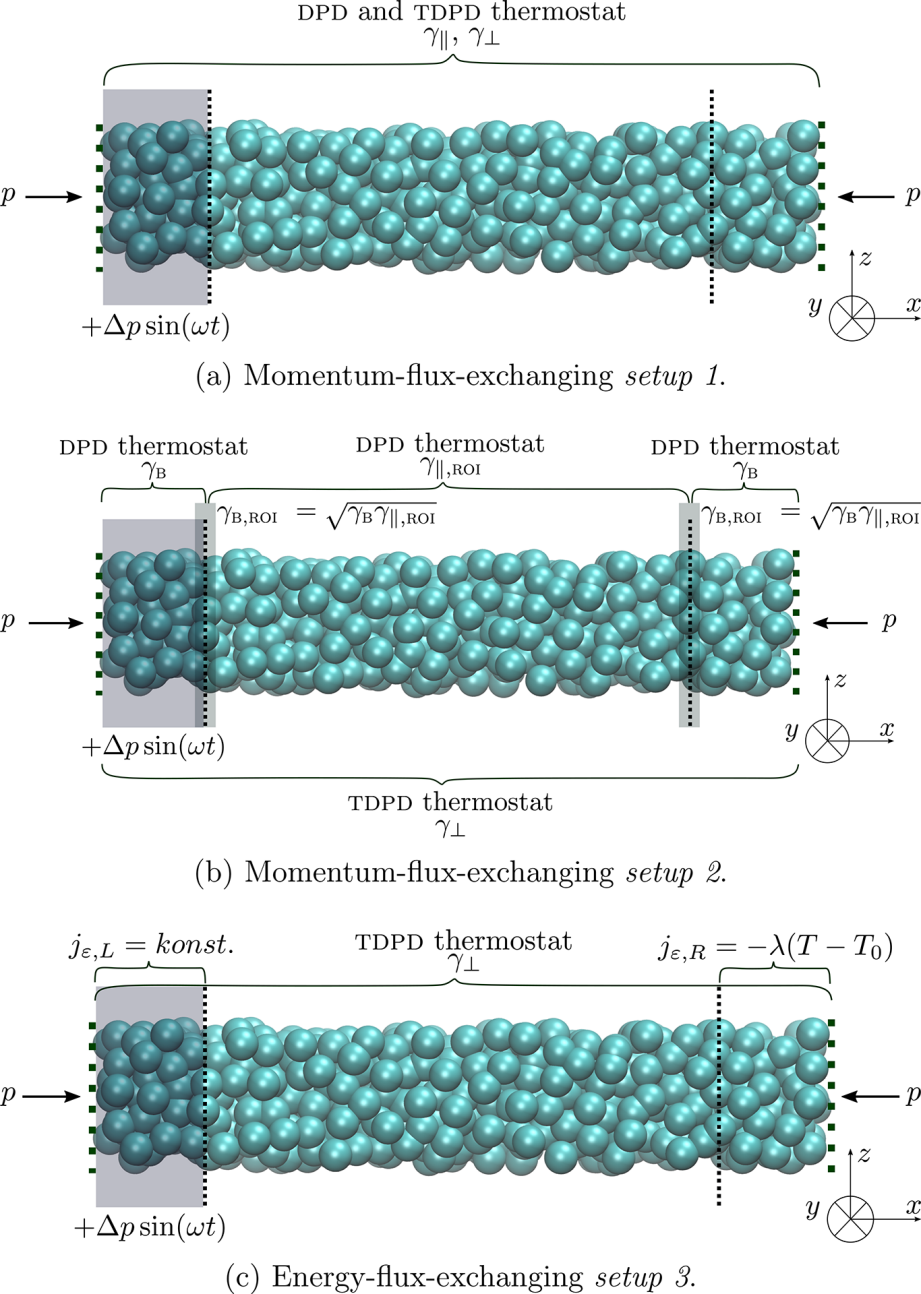
Schematic representations of the *setups* used in the OBMD to simulate the propagation of an ultrasound wave through a DPD water.

Three different *setups* are used to simulate ultrasound. *Setups 1* and *2* are momentum-flux-exchanging, while *setup 3* is energy-flux-exchanging. The governing equations for the implementation of both *setups 1* and *2* are eqs 17 and 20. Since the DPD thermostat is used in both *setups*, also eqs 5, 15, and 16 are valid. In momentum-flux-exchanging *setup 1*, the DPD thermostat acts on all particles within the simulation domain, that is, buffer + ROI, and the friction coefficient in the parallel direction (i.e., γ_∥_) has a constant value. To check the effect of γ_∥_ on the attenuation of ultrasound waves, different γ_∥_ are inspected for the momentum-flux-exchanging *setup 2*. The value of γ_∥_ depends on the position of a given particle. It is constant for particles inside buffers (i.e., γ_∥,B_) and it varies for particles within ROI (i.e., 0.0*M*_DPD_/τ_DPD_ ≤ γ_∥,ROI_ ≤ 4.0*M*_DPD_/τ_DPD_). If one particle is located in the buffer region and other in the ROI, it is calculated as the geometric mean of both γ_∥,B_ and γ_∥,ROI_. When the energy-flux-exchanging *setup 3* is implemented, only energy transfer contribution is considered, in which the DPD thermostat is switched off and only the TDPD thermostat is applied. Therefore, the governing equations for the implementation of this *setup* are eqs 5, 15, 16, 17, 18, 20, and 21. In all *setups*, the ROI is located in the center of the simulation box, that is, between two buffer regions (in Figure 1 depicted with black dotted lines).

The external boundary condition of a constant normal load (*p* = *P*^ext^) is applied onto the ROI through both buffer ends to keep the liquid inside the simulation domain. The ultrasound wave is generated by adding the oscillating pressure contribution Δ*p* sin(*ωt*) to one of the buffer ends (this region is indicated by a gray rectangle at the left side of the simulation domain in Figure 1). Correspondingly, the momentum flux tensor on the ultrasound wave generation (left) side is defined as *J*_*ij*_^*P*^ = (*p* + Δ*p* sin(*ωt*))δ_*ij*_, while the momentum flux tensor on the opposite side of the simulation domain is expressed as *J*_*ij*_^*P*^ = *pδ*_*ij*_.

*Setups 1* and *2* are expected to be suitable for studying isothermal systems, while *setup 3* is expected to be better suited for inspecting adiabatic systems. Performing OBMD simulations, we will test the presented *setups* to determine which one is more appropriate to perform simulations of ultrasound waves in the THz range.

**Figure 2:**
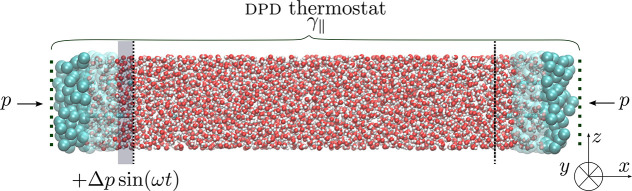
Schematic representation of the momentum-flux-exchanging *setup* used in the OBMD to simulate the propagation of an ultrasound wave through the high resolution water described by the SPC water model.

To match the viscosity of a DPD water with the viscosity of the SPC water, we couple the TDPD thermostat with the standard DPD thermostat. In contrast to simulations using soft DPD particles, in all-atom simulations, the insertion of water molecules is rather difficult due to the possible overlap with the particles of molecules already present in the system. One approach to tackle this is to use a generalized USHER algorithm.^72^ In addition to the vanilla USHER algorithm^71^ described above, in the generalized scheme, insertion of a molecule at the prescribed potential energy is achieved not only by translation, but also by rotating the molecule around its center of mass. Another way is to follow a multiscale approach,^69,76,77^ in which one uses CG-particle description to insert molecules. In this approach, the buffer region consists of three parts of different resolutions, as depicted in Figure 2. The part adjacent to the ROI is described in the high (AT) description, while the low (CG) resolution particle description is used at the open ends of the simulation box. New CG molecules are inserted into the low resolution part of the buffer, where soft intermolecular interactions are acting between CG particles. Soft intermolecular interactions can be obtained, for example, by the iterative Boltzmann inversion method (IBI).^78,79^ Inserted CG particles can freely diffuse from the low to the high resolution domain of the simulation box and acquire atomistic degrees of freedom. The bridging between different levels of description in the same simulation box is done by AdResS, as discussed next.

### 2.4 Adaptive Resolution Scheme (AdResS)

AdResS^80−82^ is a scheme that concurrently couples different levels of molecular description in the same simulation box. The latter is divided into three regions, AT, CG, and HY regions. The AT region is located in the center of the simulation box. In this region, molecules are described in high resolution. On the other hand, low resolution molecules are present in the CG regions, which are located at the ends of the simulation box. The smooth transition between the low and high resolution level of description (and *vice versa*) takes place in the HY region, which is located between the AT and CG regions. The total intermolecular force acting between two molecules α and β is
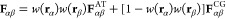
22where
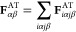
23and
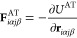
24**F**_*αβ*_^AT^ is the AT contribution to the intermolecular force and *U*^AT^ represents the intermolecular potential between AT particles. The CG contribution to the intermolecular force is
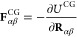
25where *U*^CG^ stands for the intermolecular potential between CG particles. The vector **r**_*iαjβ*_ = **r**_*iα*_ – **r**_*jβ*_ is the relative position vector of atoms *i* in molecule α and atoms *j* in molecule β. **R**_*αβ*_ = **R**_α_ – **R**_β_ is the relative position vector of centers of mass of molecules α and β. *w* is the position dependent weighting function. For molecules within the AT region, ω is equal to 1, whereas for molecules within the CG region it is equal to 0. In the HY region, it changes its value from one to another. The total force between two molecules α and β obeys Newton’s third law (**F**_*αβ*_ = −**F**_*βα*_) and like DPD conserves the linear momentum.^80,81^ However, the force-based AdResS does not conserve energy, and the force defined in eq 22 is in general not conservative in the transition region. Consequently, to supply or remove the latent heat associated with the change of level of description, a locally acting thermostat is required, the forces of which are just added to the scheme.^82,83^ Here, the DPD thermostat is used (see section 2.2).

## 3 Computational Details

### 3.1 DPD Details

For the mesoscopic DPD water model, we choose an 8-to-1 mapping scheme.^84^ Therefore, the coarse-graining parameter *N*_*m*_, representing the number of water molecules in one DPD particle, is 8. The mass of one DPD particle *M*_DPD_ corresponds to *m*_*i*_*N*_*m*_, where *m*_*i*_ stands for a mass of one water molecule. The DPD number density ρ̅, defined as the number of beads contained in a cube of volume *R*_*c*_^3^, is set to 3. The physical length scale is set by , where ρ_*w*_(*T*) stands for the density of liquid water (in g/cm^3^) at a temperature *T*. The unit of time is set by . The physical scale for force in a single-component system is set by the repulsion parameter *a̅*_*ij*_ and is usually defined by *a̅*_*ij*_ = (*N*_*m*_κ_exp_^–1^ – 1)/2αρ̅ (in units of *k*_B_*T*/*R*_*c*_). By adjusting the repulsion parameter, the experimental compressibility κ of the system is reproduced. Liquid water at room temperature has a compressibility of κ_exp_^–1^ ≈ 16. The friction coefficient in the parallel direction is set with .^55,84,85^ Usually, the equations of motion are integrated using a modified velocity Verlet algorithm,^62^ but in this work, the standard velocity Verlet algorithm is used and a time step of 0.001τ_DPD_. The energy scale is given by ε_DPD_ = *k*_B_*T*.

### 3.2 Atomistic Simulation Details

In OBMD simulations, atomistic SPC water molecules^86^ are only present in the ROI, while low resolution particles representing one water molecule are present in the CG region. The choice of using the SPC water model was motivated by performing hybrid simulations, where for coupling to supramolecular water models (e.g., MARTINI and DPD) bundled water models formed by introducing half-harmonic bonds between atomistic SPC waters are employed.^84,87−89^ We simulate the water system at ambient conditions, that is, at a temperature of 300 K and density 998 kg/m^3^. The intermolecular interactions are described with the Lennard-Jones potential. The cutoff distance for the nonbonded interactions is 2.84σ, and they are capped at 0.54σ, 0.25σ, and 0.44σ for oxygen–oxygen, oxygen–hydrogen, and hydrogen–hydrogen interactions, respectively, where σ stands for the length scale. The cutoff distance for the DPD thermostat equals to the cutoff distance of the nonbonded interactions. The reaction field method is used for the electrostatic interactions beyond the cutoff. The dielectric permittivity is set to 1 and 80 for the inner and outer regions, respectively. The geometry of the water molecules is constrained using SETTLE.^90^ Soft intermolecular interactions between CG particles are obtained by the IBI method.^78,79^ The conservative force in the DPD equations of motion (see eq 5) is replaced by the force derived from the atomistic force field, where dissipative and random forces remain the same (application of the DPD thermostat^64,65^). The equations of motion are integrated using the velocity Verlet algorithm^91^ and a time step of 0.0006τ_MD_, where τ_MD_ represents a unit of time. Mass and energy scale is given by *M*_MD_ and ε_MD_, respectively.

### 3.3 Computing Temperature Profile, Speed of Sound, and Attenuation Coefficient from Ultrasound Simulation

Simulations of length 500 × *t*_0_ are performed, where *t*_0_ stands for a time needed for one oscillation, and the last 400 × *t*_0_ is used for the production run. In addition, to simulate ultrasound waves of different frequencies, we need to resort to different sizes of simulation boxes due to the reflection of ultrasound waves with lower frequency at the boundary between buffer and ROI. These spurious reflections can also be overcome by using nonreflecting boundary conditions.^92^

After every ultrasound simulation, the temperature profile through the simulation box is computed. Accordingly, the simulation box is divided into *n*_*x*_ bins in the direction of the ultrasound propagation (i.e., *x*-direction), where the number of bins depends on the size of the simulation box. The temperature in each bin is calculated by the equipartition theorem, followed by averaging over the simulation time.

To determine the speed of sound and attenuation coefficient, density signals over time are first calculated. To obtain density signals over time, the ROI is divided into *n*_*x*_ × *n*_*y*_ × *n*_*z*_ cells in the *x*-, *y*-, and *z*-directions, where the number of cells depends on the size of the ROI. The density is calculated for each cell and space-averaged in the *y*–*z* plane corresponding to the homogeneous directions. Additionally, the trajectory is divided into time intervals corresponding to *t*_0_, followed by phase averaging. For each cell in the open direction (i.e., in the direction of the propagating ultrasound wave), the density signal over time *t*_0_ is computed.

Afterward, for each cell (i.e., at different distances), the parameter *k* is computed using ρ(*x*,*t*) = ρ_0_ + ρ̅ sin(*ωt* – *kx* + φ), where ω, *x*, and φ are known input parameters. Quantity ρ_0_ stands for the unattenuated amplitude of the propagating ultrasound wave, and ρ̅ stands for the amplitude. The parameter *k* is at first free in order to determine the best value. The speed of sound is determined from the best fitting parameter *k*, which is used again in the above equation to determine ρ̅ for each cell. The attenuation coefficient is finally calculated using ρ̅(*x*) = *be*^–*αx*^.

## 4 Results and Discussion

In water, ultrasound waves with the frequency in the MHz range travel the distance of several centimeters or even meters. In contrast, ultrasound waves in the THz range are absorbed on a very short distance.^38,51,66,74,93^ Nevertheless, we focus here on simulations of ultrasound waves in the THz range because our future applications will be concerned with excitation of the low-frequency vibrational modes in biomolecules.^94−97^

We perform molecular simulations both in and out of thermodynamic equilibrium using the OBMD method. Since our primary interest is the propagation of ultrasound waves we first determine the equation of state (EOS) and calculate the speed of sound for the SPC and DPD water models from equilibrium simulations at different constant normal loads (i.e., different *p* = *P*^ext^). We then determine the viscosity of simulated systems from simulations under shear flow at different strengths.^69^ We present computed properties in Table 1 together with the experimentally obtained data. The computed viscosity of the SPC water model is close to the experimentally determined viscosity for water at 25 °C. For comparison, Smith et al.,^98^ by performing equilibrium MD simulations, and Song et al.,^99^ by nonequilibrium MD, determined that the viscosity of the SPC water model is approximately 0.5 mPa s. The approximately two times higher viscosity is due to different thermostats used (Berendsen vs DPD) in these works. The speed of sound determined from the EOS of the SPC water corresponds to the experimentally determined speed of sound for water. The viscosity of the DPD water can be matched to the viscosity of the simulated SPC water by applying the TDPD thermostat in DPD simulations. Conveniently, a speed of sound computed from the EOS for the DPD water corresponds to the speed of sound for the SPC water.

**Table 1:** Computed Properties with Associated Standard Deviations and Parameters Used in Simulations of SPC and DPD Water, and Experimentally Determined Properties for Water at 25 °C

model	γ_∥_	γ_⊥_	η	*c*
SPC	0.049 [*M*_MD_/τ_MD_]	0.0 [*M*_MD_/τ_MD_]	17.60 ± 0.06 [ε_MD_τ_MD_/σ^3^]	7 ± 2 [σ/τ_MD_]
			0.940 ± 0.003 [10^–3^ Pa s]	1487 ± 640 [m/s]
DPD	4.5 [*M*_DPD_/τ_DPD_]	1.5 [*M*_DPD_/τ_DPD_]	23.3 ± 0.3 [ε_DPD_τ_DPD_/*R*_*c*_^3^]	11.4 ± 0.1 [*R*_*c*_/τ_DPD_]
Experiment			0.890^100^ [10^–3^ Pa s]	1479^100^ [m/s]

As a representative set of ultrasound frequencies in the THz range, we choose six different frequencies which are further divided into low (0.92τ_DPD_^–1^, 0.46τ_DPD_^–1^, 0.27τ_DPD_^–1^) and high (2.76τ_DPD_^–1^, 2.15τ_DPD_^–1^, 1.84τ_DPD_^–1^). To investigate the ultrasound properties, that is, the attenuation and speed of sound, of the simulated ultrasound waves, we use three boxes of different dimensions, 30 × 10 × 10*R*_*c*_^3^, 130 × 5 × 5*R*_*c*_^3^, and 260 × 5 × 5*R*_*c*_^3^ in the *x*-, *y*-, and *z*-directions. Corresponding ROI sizes are *x*_ROI,1_ = 21*R*_*c*_, *x*_ROI,2_ = 91*R*_*c*_, and *x*_ROI,3_ = 182*R*_*c*_ in the case of the smallest, middle, and largest simulation box used, respectively. By implementing the momentum-flux-exchanging *setup 1* (see Figure 1a), where the DPD thermostat acts on all particles within the simulation domain, that is, buffers+ROI, the effect of a frequency on the attenuation and speed of sound is examined.

**Figure 3:**
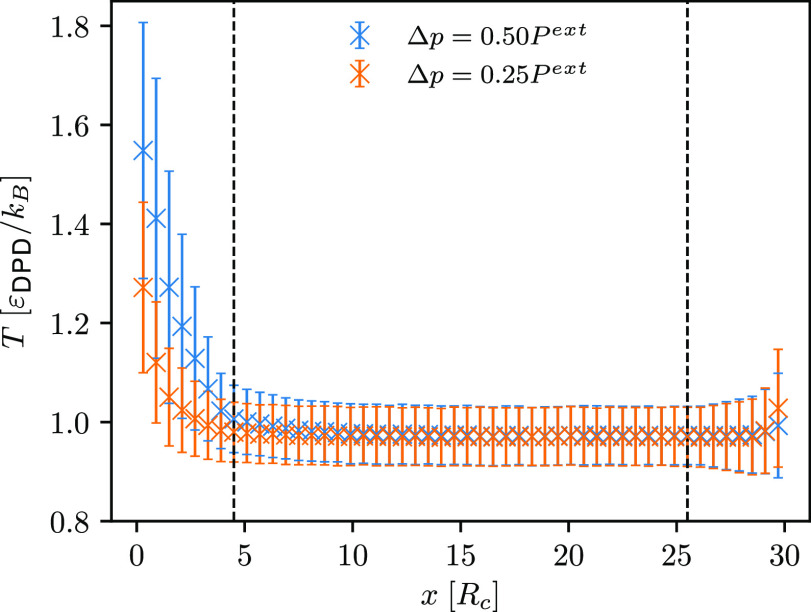
Computed temperature profiles through the simulation box (*setup 1*) for the ultrasound wave with a frequency of ν = 1.84τ_DPD_^–1^ and two different amplitudes. Colored crosses indicate average temperature, while error bars denote the associated standard deviation.

**Figure 4:**
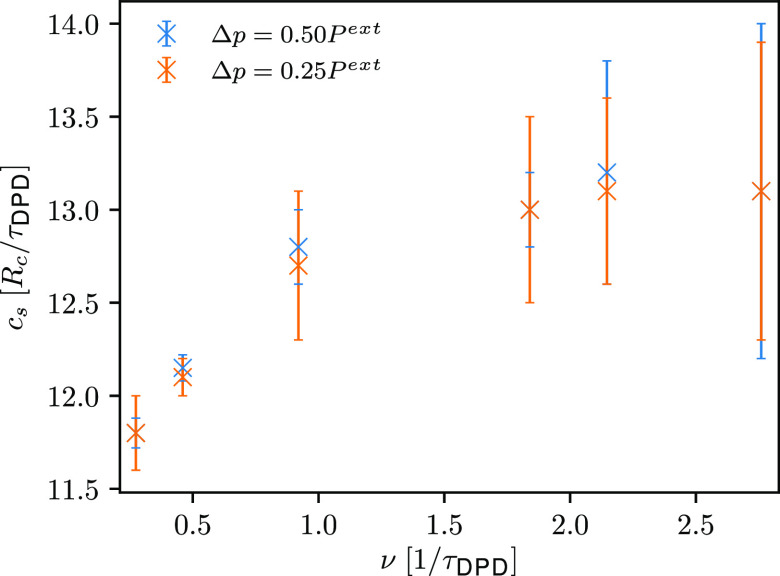
Comparison of the computed speed of sound with associated standard deviations, represented with error bars, for ultrasound waves of different frequencies and two different amplitudes (*setup 1*).

To check if the temperature profiles are flat and at the expected temperature, we compute them (as discussed in section 3.3) and depict one in Figure 3 (temperature profiles for ultrasound waves with a frequency of 2.76τ_DPD_^–1^, 2.15τ_DPD_^–1^, 0.92τ_DPD_^–1^, 0.46τ_DPD_^–1^, and 0.27τ_DPD_^–1^ are shown in Figure S1 in the Supporting Information). Because the temperature profiles are indeed flat, we anticipate that the computed speed of sound (*c*_s_) will be comparable to the one determined from the EOS of a DPD water (see Table 1). Following the procedure described in section 3.3, we observe a linear increase in the calculated speed of sound with increasing frequency for the low frequency ultrasound waves. However, this is not the case for the high frequency ultrasound waves, since computed values of speed of sound are about the same (see Figure 4). For gases, the speed of sound should approach the adiabatic speed of sound for lower frequencies, while for higher frequencies it should approach the isothermal speed of sound.^43^ As evident from Figure 4, with decreasing frequency the computed speed of sound is getting closer to the one determined from the EOS of a DPD water (see Table 1) and therefore, ultrasound waves can be considered as isothermal. Using the computed speed of sound, we can calculate the wavelengths of simulated ultrasound waves. The corresponding wavelengths for the ultrasound waves with a frequency of 2.76τ_DPD_^–1^, 2.15τ_DPD_^–1^, and 1.84τ_DPD_^–1^ are 0.25*x*_ROI,1_, 0.30*x*_ROI,1_, 0.34*x*_ROI,1_, respectively, while for the ultrasound waves with a frequency of 0.92τ_DPD_^–1^, 0.46τ_DPD_^–1^, and 0.27τ_DPD_^–1^ they are 0.15*x*_ROI,2_, 0.15*x*_ROI,3_, and 0.25*x*_ROI,3_, respectively.

**Figure 5:**
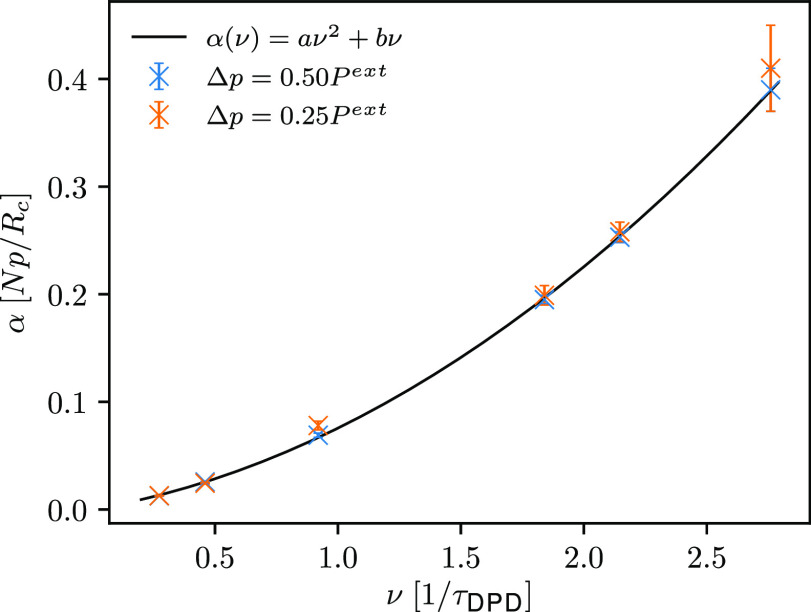
Comparison of the computed attenuation coefficients with associated standard deviations, represented with error bars, for ultrasound waves of different frequencies and two different amplitudes (*setup 1*). Crosses indicate results calculated from simulations using OBMD, while the black line corresponds to the quadratic dependence of the attenuation coefficients on the frequency for ultrasound waves with an amplitude of 0.50*P*^ext^, where *a* = (0.0372 ± 0.0007)*Npτ*_DPD_^2^/*R*_*c*_ and *b* = (0.038 ± 0.001)*Npτ*_DPD_^2^/*R*_*c*_.

Interestingly, the attenuation coefficients are observed to increase quadratically with increasing frequency of ultrasound waves (see Figure 5). Similarly, taking propagation of acoustic waves in air, Fletcher^51^ also proposed a quadratic increase of attenuation coefficients with increasing frequency.

**Figure 6:**
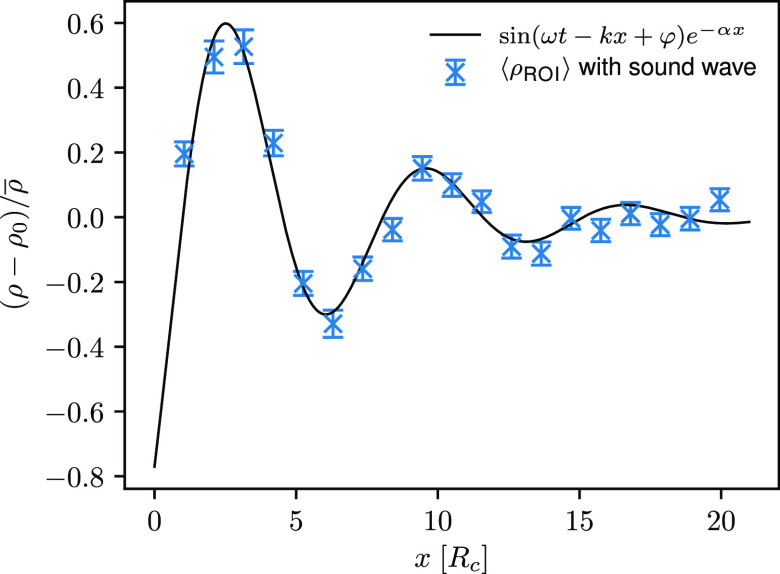
Computed density signal through the ROI for the ultrasound wave with a frequency of 1.84τ_DPD_^–1^ and an amplitude of 0.50*P*^ext^ at time *t* = *t*_0_ (*setup 1*). Blue crosses indicate results calculated from simulation using OBMD, error bars represent the associated standard error, and the black line corresponds to the analytical solution.

In addition, as shown in Figure 6, we observe a good agreement of the calculated density signals with the analytical solutions (corresponding to the solution of the wave equation described in section 2.1). Computed density signals for ultrasound waves with a frequency of 2.76τ_DPD_^–1^, 2.15τ_DPD_^–1^, 0.92τ_DPD_^–1^, 0.46τ_DPD_^–1^, and 0.27τ_DPD_^–1^ are depicted in Figure S5.

Assuming that the friction coefficient γ_∥_ affects the propagation of ultrasound waves, its effect is studied by implementing the momentum-flux-exchanging *setup 2*. Accordingly, the value of a friction coefficient through the ROI γ_∥,ROI_ is varied between 0.0*M*_DPD_/τ_DPD_ and 4.0*M*_DPD_/τ_DPD_, while in the buffer regions it is constant. With this *setup* (see Figure 1b) and simulation box of size 30 × 10 × 10*R*_*c*_^3^ in the *x*-, *y*-, and *z*-directions, only the high frequency ultrasound waves are simulated, since these ultrasound waves are also the most attenuated. Corresponding ROI size is *x*_ROI_ = 21*R*_*c*_. As for *setup 1*, we get flat temperature profiles through the ROI at the expected temperature regardless of the friction coefficient used (see Figure S2).

**Figure 7:**
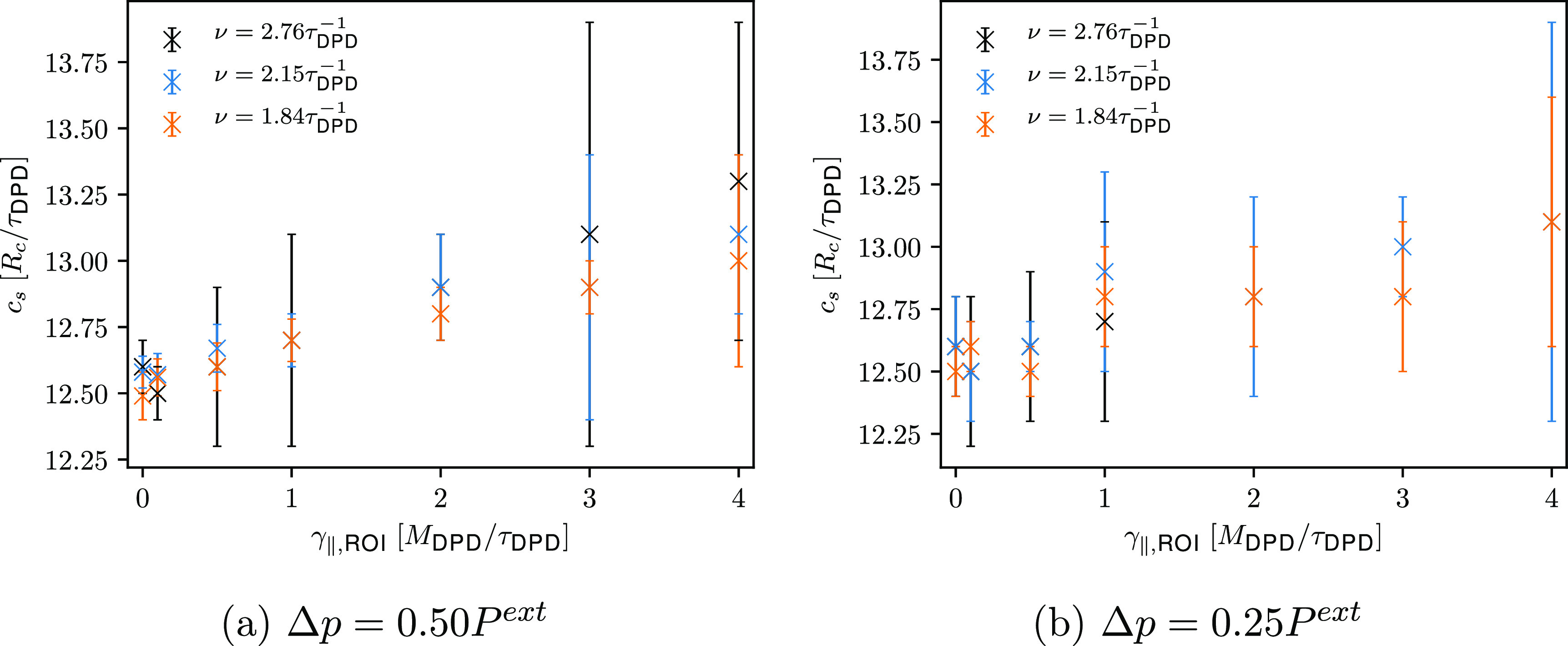
Comparison of the computed speed of sound with respect to the friction coefficients γ_∥,ROI_ used for simulation of ultrasound waves of different frequencies and two different amplitudes (*setup 2*).

Although γ_∥,ROI_ does not affect the temperature profile, it does affect the computed speed of sound. A slight linear increase of speed of sound with increasing γ_∥,ROI_ is indicated (see Figure 7). In contrast to *setup 1*, a different speed of sound is computed for different γ_∥,ROI_, which leads to slightly shorter wavelengths of the simulated ultrasound waves. Intriguingly, with decreasing γ_∥,ROI_, the computed speed of sound approaches the one determined from the EOS of a DPD water (see Table 1). Accordingly, ultrasound waves are considered as isothermal.

**Figure 8:**
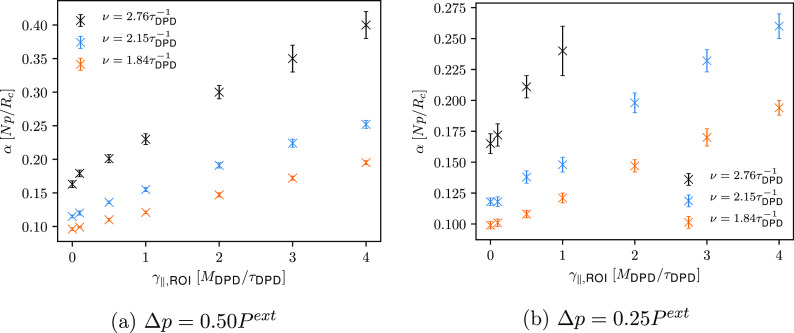
Comparison of the computed attenuation coefficients (*setup 2*).

**Figure 9:**
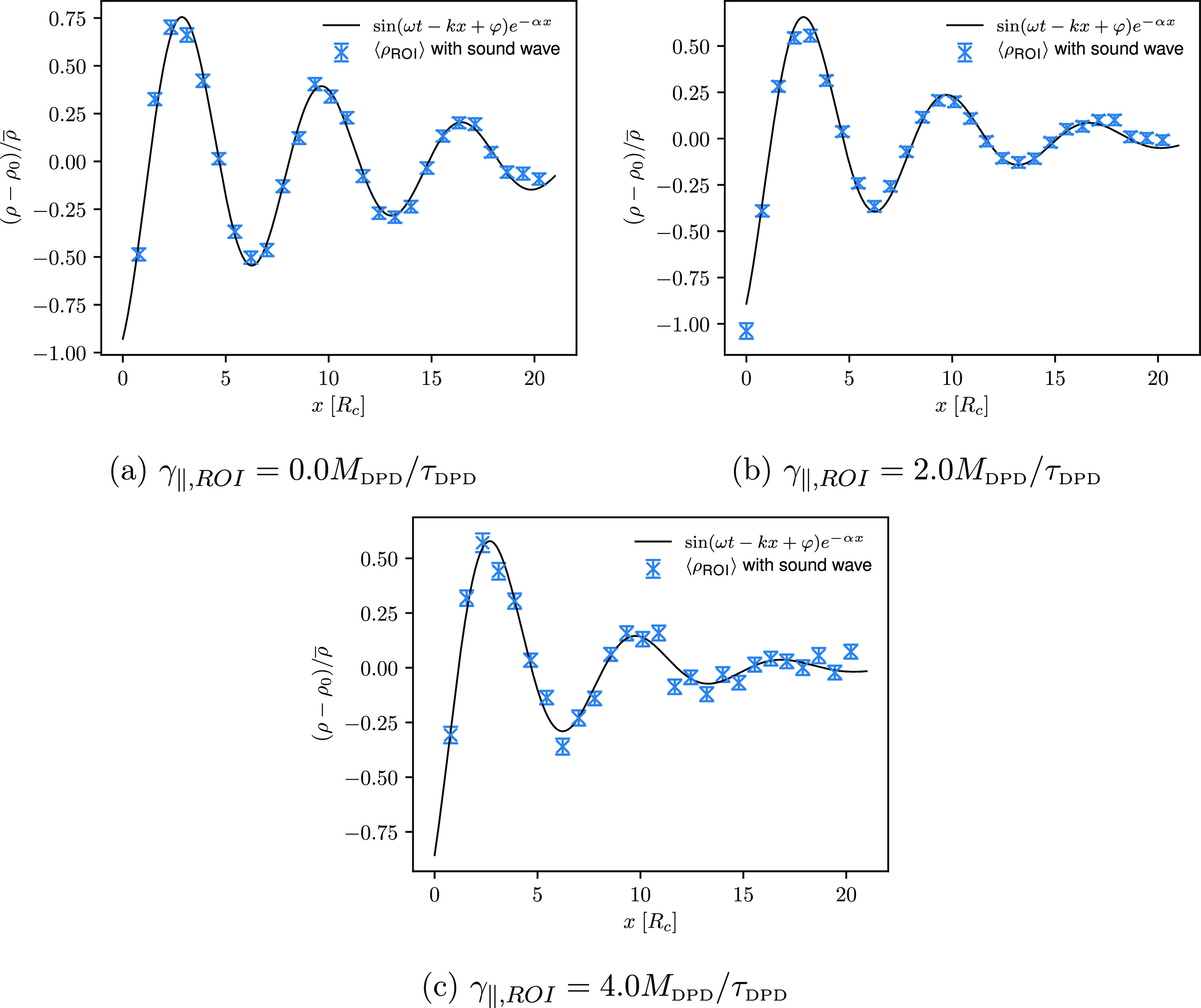
Computed density signals through the ROI for the ultrasound wave with a frequency of 1.84τ_DPD_^–1^ and an amplitude of 0.50*P*^ext^ at time *t* = *t*_0_ and for three different friction coefficients γ_∥,ROI_ used (*setup 2*).

As it turns out, γ_∥,ROI_ greatly affects the attenuation of ultrasound waves, as depicted in Figure 8. Attenuation coefficients also appear to increase linearly with increasing γ_∥,ROI_ and, as evident from Figure 8, this increase is faster for the ultrasound wave with the highest frequency. To show the influence of the selected γ_∥,ROI_ on the propagation of ultrasound waves, calculated density signals through the ROI for the ultrasound wave with a frequency of 1.84τ_DPD_^–1^ are shown in Figure 9. Similar to *setup 1*, we observe a good agreement between the calculated density signals and the analytical solutions regardless of the friction coefficient γ_∥,ROI_ used. Computed density signals for the ultrasound waves with a frequency of 2.76τ_DPD_^–1^ and 2.15τ_DPD_^–1^ are shown in Figures S6, S7, and S8 in the Supporting Information.

The observed influence of the selected γ_∥,ROI_ on the computed properties inspires us to implement the momentum-flux-exchanging *setup 3*, as it allows control of the energy flux without the use of a thermostat. Therefore, the DPD thermostat is completely switched off. We choose the same simulation box size as in *setup 1*, since we simulate ultrasound waves of the same frequencies. Here, we show results for ultrasound waves with an amplitude of 0.25*P*^ext^, while at higher pressure amplitude the control of the energy transfer contribution (defined by eq 21) is rather challenging. As for *setups 1* and *2*, we observe flat temperature profiles through the ROI at the expected temperature (see Figure S3 in the Supporting Information).

**Figure 10:**
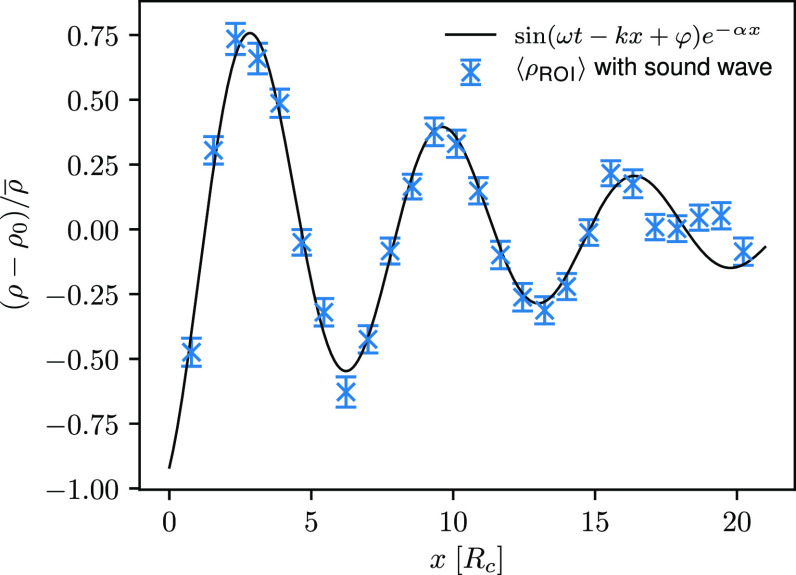
Computed density signal through the ROI for the ultrasound wave with a frequency of 1.84τ_DPD_^–1^ and an amplitude of 0.25*P*^ext^ at time *t* = *t*_0_ (*setup 3*).

In contrast to the momentum-flux-exchanging *setups 1* and *2*, using the energy-flux-exchanging *setup 3* indicates that the calculated speed of sound does not depend on the frequency of ultrasound waves (see Table 2). As for *setup 2*, we observe slightly shorter wavelengths. Nevertheless, the computed values of speed of sound are close to the one determined from the EOS of a DPD water (see Table 1) and therefore, ultrasound waves are still considered as isothermal. As expected, we also observe that the attenuation of ultrasound waves with increasing frequency increases. Despite using another approach, we observe a good agreement between the computed density profiles and the analytical solutions, as shown in Figure 10 for the ultrasound wave with a frequency of 1.84τ_DPD_^–1^. Calculated density signals for the ultrasound waves with a frequency of 2.76τ_DPD_^–1^ and 2.15τ_DPD_^–1^ are depicted in Figure S9 in the Supporting Information.

**Table 2:** Computed Speed of Sound and Attenuation Coefficients for Ultrasound Waves of Different Frequencies and an Amplitude of 0.25*P*^ext^ (*setup 3*)

ν[1/τ_DPD_]	*c*_*s*_[*R*_*c*_/τ_DPD_]	α[*Np*/*R*_*c*_]
2.76	12.4 ± 0.3	0.158 ± 0.007
2.15	12.4 ± 0.1	0.115 ± 0.003
1.84	12.4 ± 0.1	0.096 ± 0.003

**Figure 11:**
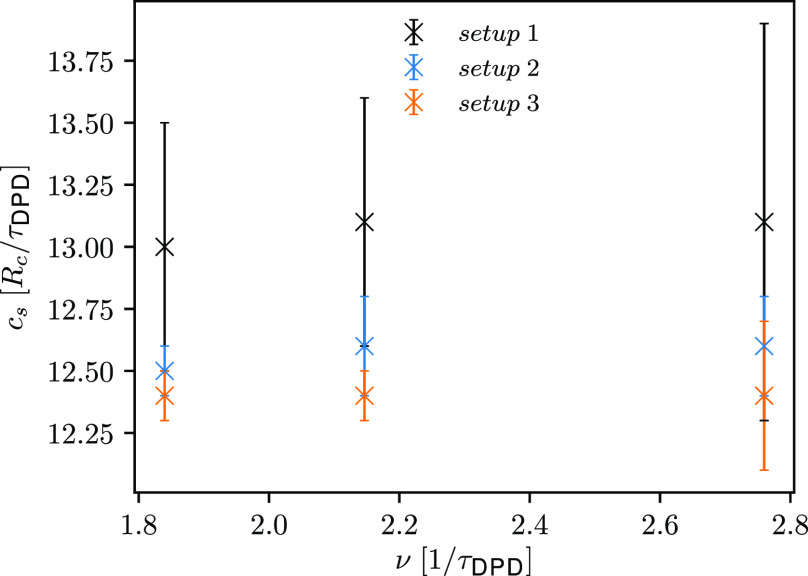
Comparison of the computed speed of sound with respect to the *setups* used for ultrasound waves of different frequencies and an amplitude of 0.25*P*^ext^.

Finally, let us comment on the performance of the *setups 1*, *2*, and *3* for simulations of ultrasound waves in the THz range. To this end, we compare the results where the friction coefficient γ_∥,ROI_ = 0.0*M*_DPD_/τ_DPD_ for *setup 2*. We expect that the determined speed of sound for ultrasound waves simulated using *setups 2* and *3* will be comparable. For the ultrasound waves simulated using *setup 1*, we compute the highest values of speed of sound, while the calculated values of speed of sound are within error bars for *setups 2* and *3*, as depicted in Figure 11. Note that as γ_∥,ROI_ increases (*setup 2*), the values of speed of sound approach the speed of sound determined for the *setup 1*. This is not surprising, since the momentum-flux-exchanging *setup 2* is another variant of the momentum-flux-exchanging *setup 1*, where for the latter a constant γ_∥_ is used through the simulation domain (see Table 1).

**Figure 12:**
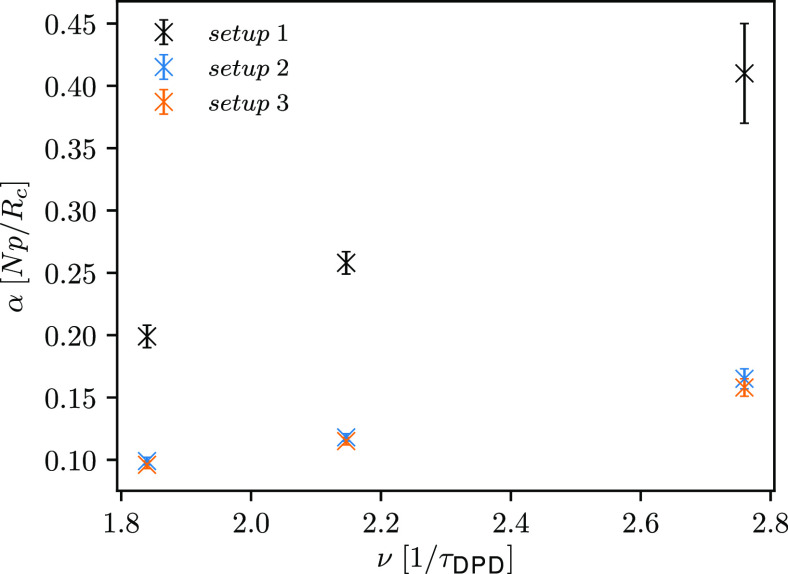
Comparison of the computed attenuation coefficients for different *setups*.

For the same reason, we anticipate that the computed attenuation coefficients for ultrasound waves simulated using *setups 2* and *3* will also be comparable. As shown in Figure 12, the computed attenuation coefficients are within error bars, while the ultrasound waves simulated employing *setup 1* are the most attenuated. Similar as in the case of the speed of sound, with increasing γ_∥,ROI_ (*setup 2*) the attenuation of ultrasound waves increases.

The results in Figure 12 indicate that the attenuation of ultrasound waves can be reduced by using smaller friction coefficients when applying the momentum-flux-exchanging *setup 2* or simply by implementing the energy-flux-exchanging *setup 3*. For all *setups*, we observe a good agreement between the computed density signals and analytical solutions. However, using different *setups*, one needs to be aware of the influence of the selected friction coefficients (e.g., γ_∥_, γ_∥,ROI_, and γ_⊥_) and also other parameters on the physical properties (for example, viscosity) of the simulated system.

Because in our future work the presented virtual ultrasound machine will be used to excite the low-frequency vibrational modes of proteins in water, we also test it to propagate ultrasound waves through the atomistic water. For this purpose, we use the momentum-flux-exchanging *setup*, where the DPD thermostat acts on all particles within the simulation domain (i.e., buffers+ROI), combined with AdResS (see Figure 2). We choose to simulate ultrasound waves with a frequency of 0.31τ_MD_^–1^ and 0.21τ_MD_^–1^, while they belong to the frequency range corresponding to the vibrational modes in proteins. Using a simulation box of size 49.96 × 8.85 × 8.85σ^3^ in the *x*-, *y*-, and *z*-directions, we simulate ultrasound waves with a frequency of 0.31τ_MD_^–1^, and while using a simulation box of size 99.93 × 8.85 × 8.85σ^3^, we simulate ultrasound waves with a frequency of 0.21τ_MD_^–1^. Corresponding ROI sizes are *x*_ROI,1_ = 35.40σ in the case of a smaller simulation box and *x*_ROI,2_ = 85.37σ in the case of a larger simulation box. We simulate ultrasound waves with different pressure amplitudes, but only those with higher amplitude (i.e., Δ*p* = 1.3*P*^ext^ and 2.0*P*^ext^) are suitable for our analysis due to very noisy signals obtained for ultrasound waves with lower pressure amplitudes. As for simulations employing the DPD water model and implementing different *setups*, we observe flat temperature profiles through the ROI at the expected temperature (see Figure S4 in the Supporting Information).

To compute the speed of sound, we follow the same procedure as in DPD simulations. We observe that with decreasing frequency, the calculated speed of sound approaches to the one determined from the EOS of the SPC water (see Tables 2 and 1). Therefore, simulated ultrasound waves can be considered as isothermal. Using the computed speed of sound, we calculate the corresponding wavelengths; that is, for the ultrasound wave with a frequency of 0.31τ_MD_^–1^ it is approximately *x*_ROI,1_, while for the ultrasound wave with a frequency of 0.21τ_MD_^–1^ it is equal to 0.55*x*_ROI,2_.

As expected, we observe that the high frequency ultrasound wave is more attenuated (see Table 3). Interestingly, even though we matched the viscosity of DPD to the reference value of the atomistic water, nevertheless, slightly higher attenuation coefficients are determined for the atomistic system. Similarly, this applies also to the computed speed of sound. We attribute this disagreement to softer interactions between DPD waters in comparison to atomistic water. As for DPD simulations, computed density signals are in a good agreement with the analytical solutions (see Figure S10 in the Supporting Information).

**Table 3:** Computed Speed of Sound and Attenuation Coefficients with Associated Standard Deviations for Ultrasound Waves of Different Frequencies and Two Different Amplitudes (AdResS Simulation)

ν[1/τ_MD_]	Δ*p*	*c*_*s*_[σ/τ_MD_]	α[*Np*/σ]
0.31	2.0*P*^ext^	11.6 ± 1.0	0.080 ± 0.006
	1.3*P*^ext^	11.6 ± 1.4	0.081 ± 0.006
0.21	2.0*P*^ext^	10.6 ± 0.8	0.054 ± 0.001
	1.3*P*^ext^	9.9 ± 0.8	0.047 ± 0.003

The presented virtual ultrasound machine will allow us to study different physical phenomena occurring during the ultrasound propagation.

## 5 Conclusions and Outlook

In this work, we developed the particle-based virtual ultrasound machine and tested it in simulations of ultrasound waves in the THz range using DPD and atomistic water models. The results of our particle-based ultrasound simulations show that our approach is capable of reproducing the fluctuating hydrodynamics description of ultrasound in the continuum limit. At high frequencies, sound waves in gases can be considered as adiabatic, whereas at low frequencies they can be considered as isothermal. If the frequency of a sound wave ω is comparable to the frequency associated with the thermal conduction of a medium ω_TC_, as in water, then it is not clear a priori which approximation works better. To clarify this issue, the DPD water model was used and momentum-flux-exchanging and energy-exchanging schemes were implemented and tested. Our results indicate that the isothermal classification is more appropriate. We also studied the effect of thermostat parameters on the attenuation of ultrasound waves and computed speed of sound. The greatest attenuation of ultrasound waves was observed for the momentum-flux-exchanging scheme, in which DPD and TDPD thermostats acted on all particles within simulation domain (i.e., buffer+ROI). For this scheme, we also observed a linear increase in the computed speed of sound for lower frequency ultrasound waves. Furthermore, the computed speed of sound slightly depends on the friction coefficients γ_∥,ROI_.

To conclude, the developed method enables us to study different physical phenomena associated with ultrasound-soft(bio) matter interactions. In our future work, we aim to employ the presented virtual ultrasound machine to study the low-frequency vibrational modes of biomolecules in water. Moreover, we will perform simulations of adiabatic ultrasound waves propagating in water (i.e., simulations of ultrasound waves at lower frequencies, i.e., in the GHz–MHz range, that can penetrate centimeters deep). Accordingly, larger systems will be employed and the energy-flux-exchanging approach will be implemented.

## Supporting Information Available

Computed temperature profiles and density signals for ultrasound waves of different frequencies and amplitudes simulated by OBMD (PDF)
