# Supplementary material for: Dissipative Particle Dynamics Simulation of Ultrasound Propagation through Liquid Water
Source: J Chem Theory Comput. 2022 Jan 10;18(2):1227–40. doi: 10.1021/acs.jctc.1c01020 (PMC8830050; doi:10.1021/acs.jctc.1c01020)
Supplement: Supplementary file 1 — ct1c01020_si_001.pdf [file ct1c01020_si_001.pdf]

Supporting information for

Dissipative Particle Dynamics Simulation of Ultrasound Propagation through Liquid Water

Petra Papež[1,2] and Matej Praprotnik[1,2,*]

[1]*Laboratory for Molecular Modeling, National Institute of Chemistry, Ljubljana SI-1001, Slovenia*
[2]*Department of Physics, Faculty of Mathematics and Physics, University of Ljubljana, Ljubljana SI-1000, Slovenia*

COMPUTED TEMPERATURE PROFILES FOR ULTRASOUND WAVES OF DIFFERENT FREQUENCIES AND FOR DIFFERENT *SETUPS* USED

For every *setup*, i.e., the momentum-flux-exchanging *setup 1*, momentum-flux-exchanging *setup 2*, and energy-flux-exchanging *setup 3* (for details see the main text), used, we compute temperature profiles through the region of interest (ROI) and check if they are flat and at the expected temperature.

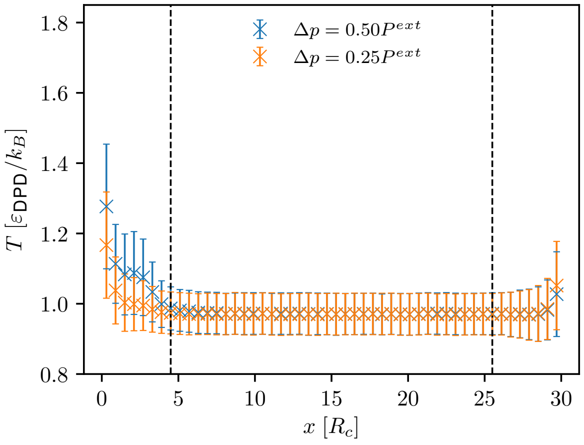

(a) $\nu = 2.76\tau_{\text{DPD}}^{-1}$

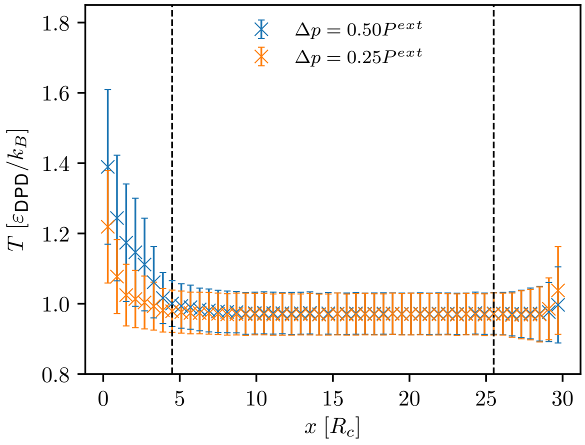

(b) $\nu = 2.15\tau_{\text{DPD}}^{-1}$

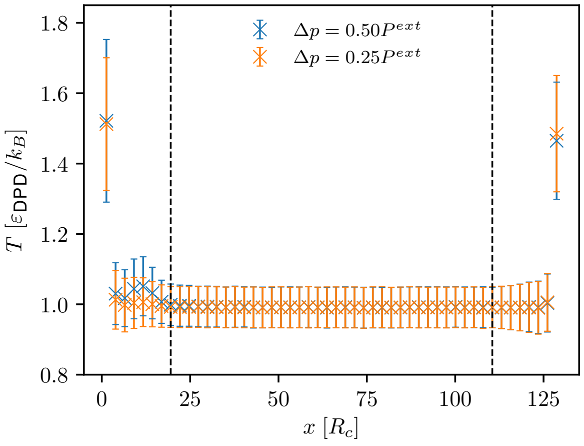

(c) $\nu = 0.92\tau_{\text{DPD}}^{-1}$

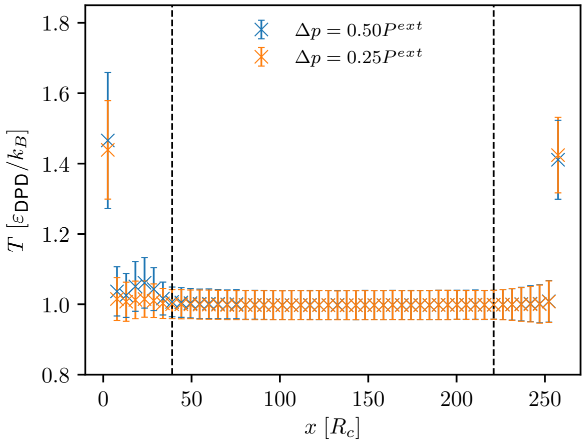

(d) $\nu = 0.46\tau_{\text{DPD}}^{-1}$

* Email: praprot@cmm.ki.si

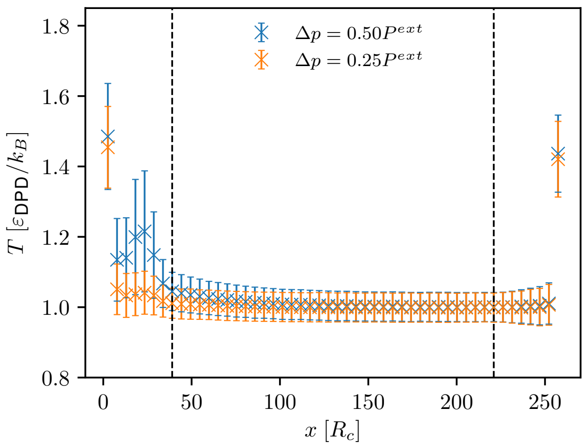

(e) $\nu = 0.27\tau_{\text{DPD}}^{-1}$

Figure S1: Computed temperature profiles through the simulation box for ultrasound waves of different frequencies and two different amplitudes, simulated by employing the momentum-flux-exchanging *setup 1*. Colored crosses indicate average temperature, while error bars denote the associated standard deviation.

Figures S1, S2, and S3 show the calculated temperature profiles for ultrasound waves simulated by implementing the momentum-flux-exchanging *setup 1* (where the DPD thermostat acts on all particles within simulation domain), momentum-flux-exchanging *setup 2* (where the value of a friction coefficient $\gamma_{\parallel,\text{ROI}}$ though the ROI is varied), and energy-flux-exchanging *setup 3* (where the DPD thermostat is switched off), respectively. For AdResS simulation, computed temperature profiles are depicted in Figure S4. As evident, calculated temperature profiles for ultrasound waves of different frequencies are flat and at the expected temperature.

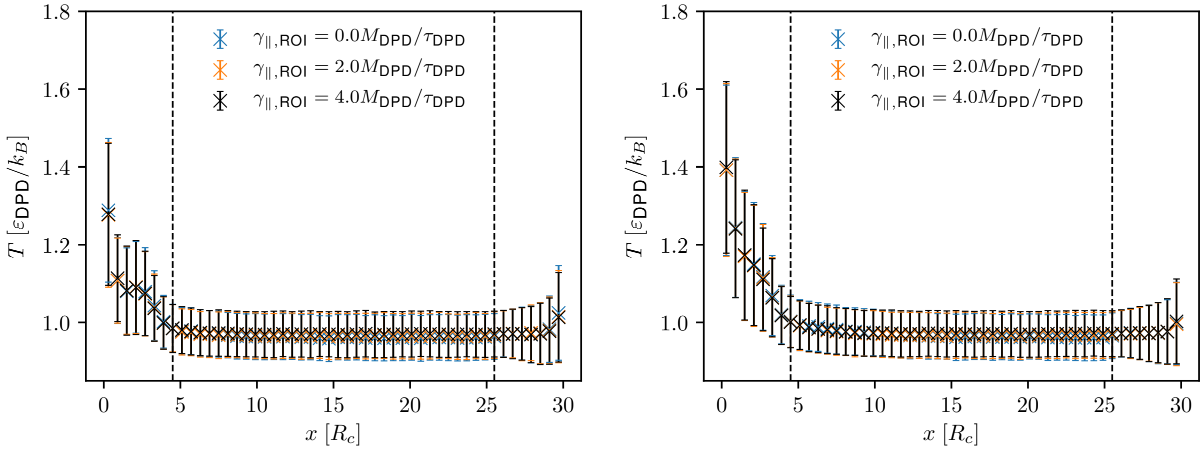

(a) $\nu = 2.76\tau_{\text{DPD}}^{-1}$

(b) $\nu = 2.15\tau_{\text{DPD}}^{-1}$

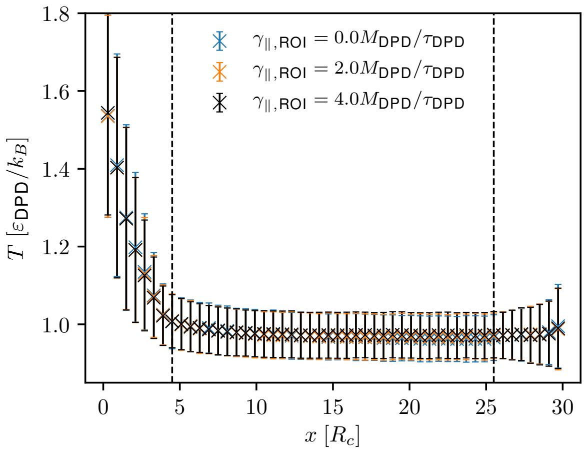

(c) $\nu = 1.84\tau_{\mathrm{DPD}}^{-1}$

Figure S2: Computed temperature profiles through the simulation box for ultrasound waves of different frequencies and an amplitude of $0.50P^{ext}$, simulated by implementing the momentum-flux-exchanging *setup 2*.

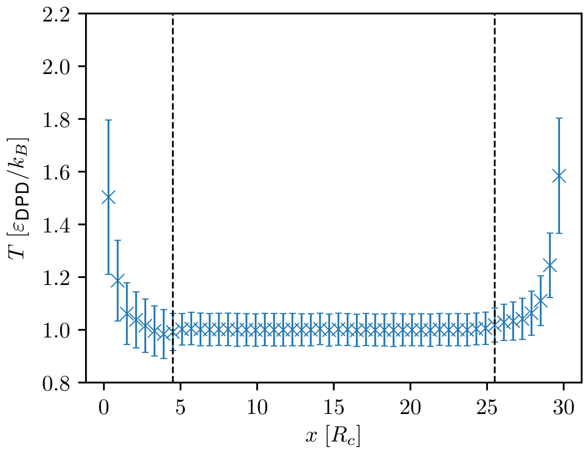

(a) $\nu = 2.76\tau_{\mathrm{DPD}}^{-1}$

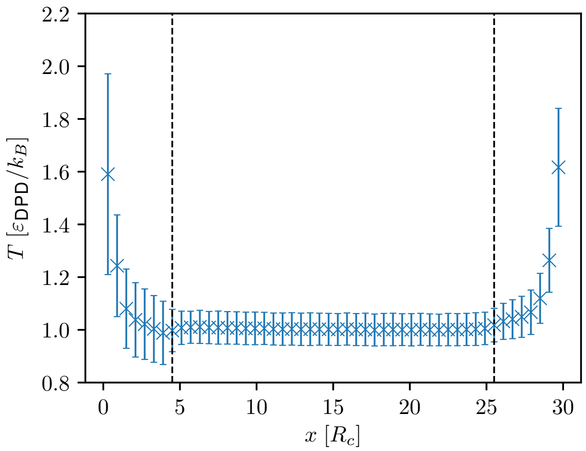

(b) $\nu = 2.15\tau_{\mathrm{DPD}}^{-1}$

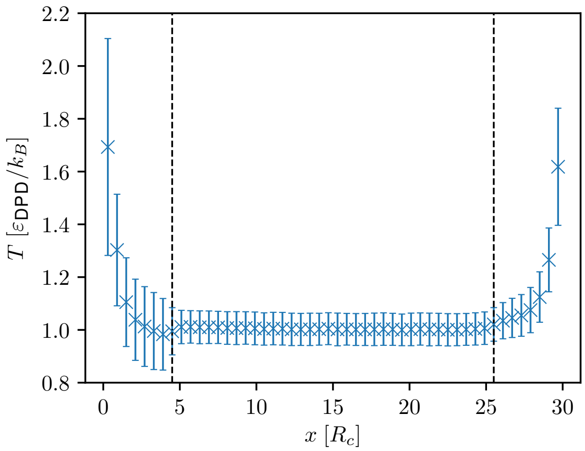

(c) $\nu = 1.84\tau_{\mathrm{DPD}}^{-1}$

Figure S3: Same as in Figure S2 for an amplitude of $0.25P^{ext}$ and energy-flux-exchanging *setup 3*.

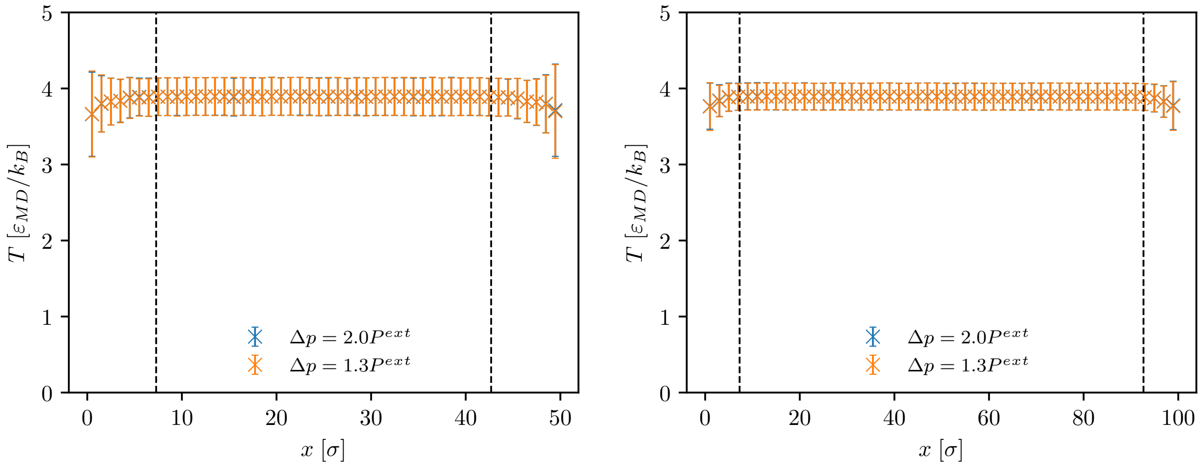

(a) $\nu = 0.31\tau_{\mathrm{MD}}^{-1}$ (b) $\nu = 0.21\tau_{\mathrm{MD}}^{-1}$

Figure S4: Same as in Figure S1 for AdResS simulation.

COMPUTED DENSITY SIGNALS FOR ULTRASOUND WAVES OF DIFFERENT FREQUENCIES AND FOR DIFFERENT *SETUPS* USED

We also check the agreement between analytical solutions and density signals calculated from the open boundary molecular dynamics (OBMD) simulations.

Figure S5 shows a good agreement between the computed density signals for ultrasound waves simulated by employing the momentum-flux-exchanging *setup 1*. As evident, lower frequency ultrasound waves are attenuated over shorter distances (for example, compare Figure S5a, i.e., high frequency ultrasound wave, with Figure S5e, i.e., low frequency ultrasound wave). A comparison of the computed density signals for ultrasound waves of different frequencies and for three different friction coefficients $\gamma_{\|,\mathrm{ROI}}$ used (i.e., for ultrasound simulations where the momentum-flux-exchanging *setup 2* is implemented) with analytical predictions is depicted in Figures S6, S7, and S8. Here, regardless of the frequency used, lower attenuation of the ultrasound waves is observed for lower $\gamma_{\|,\mathrm{ROI}}$. By conducting ultrasound simulations and implementing the energy-flux-exchanging *setup 3* (see Figure S9) and the momentum-flux-exchanging *setup 1* in combination with the adaptive resolution scheme (see Figure S10) and comparing results with the analytical solutions, we observe a good agreement between results computed from the OBMD simulations and analytical solutions.

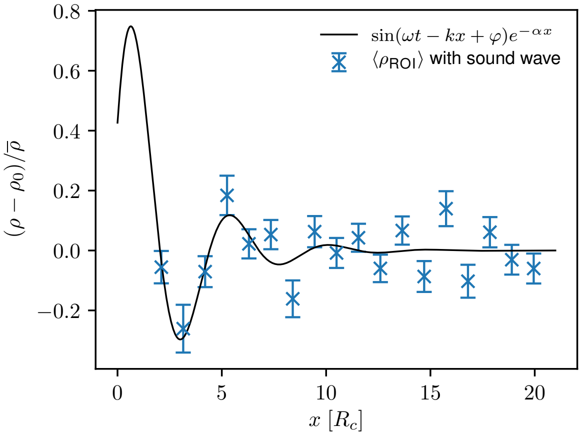

(a) $\nu = 2.76\tau_{\mathrm{DPD}}^{-1}$

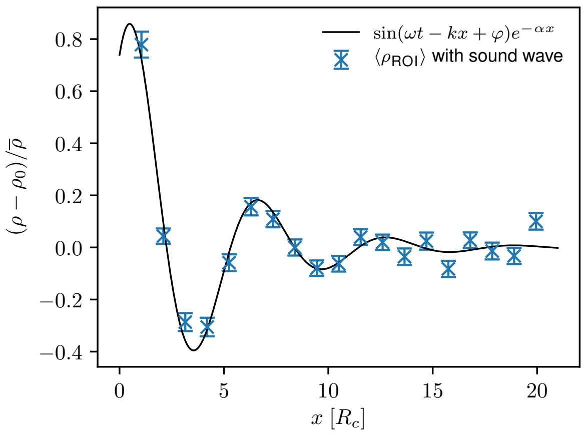

(b) $\nu = 2.15\tau_{\mathrm{DPD}}^{-1}$.

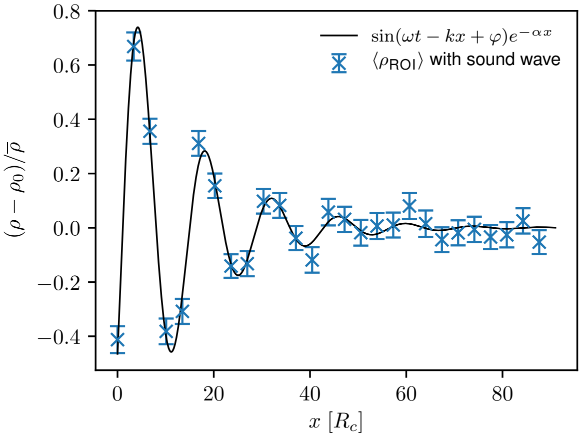

(c) $\nu = 0.92\tau_{\mathrm{DPD}}^{-1}$

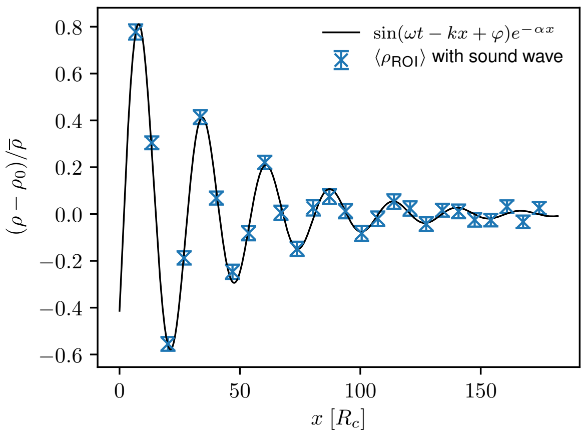

(d) $\nu = 0.46\tau_{\mathrm{DPD}}^{-1}$

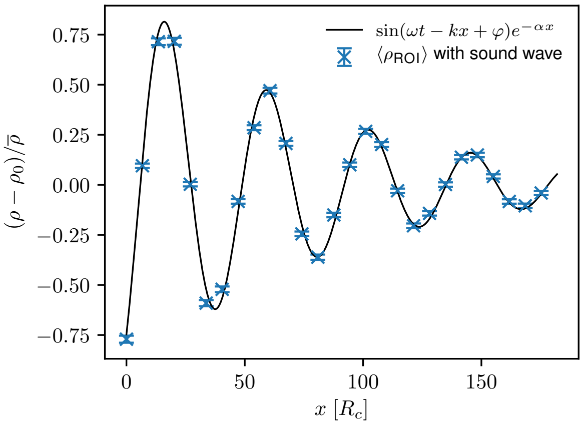

(e) $\nu = 0.27\tau_{\mathrm{DPD}}^{-1}$

Figure S5: Computed density signals through the ROI for ultrasound waves of different frequencies and an amplitude of $0.50P^{ext}$ at time $t = t_0$, simulated by employing the momentum-flux-exchanging *setup 1*. Blue crosses indicate results calculated from simulation using OBMD, error bars represent the associated standard error, and the black line corresponds to the analytical solution.

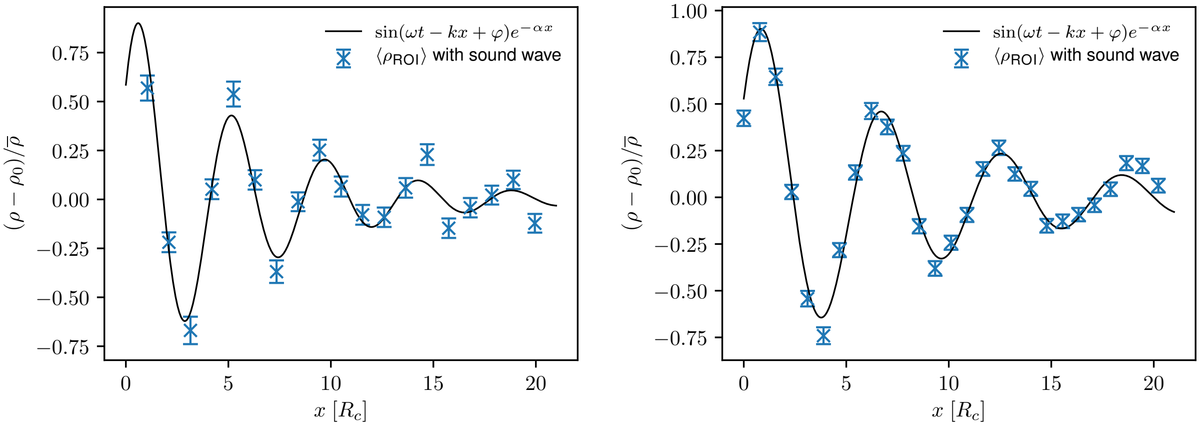

(a) $\nu = 2.76\tau_{\mathrm{DPD}}^{-1}$ (b) $\nu = 2.15\tau_{\mathrm{DPD}}^{-1}$

Figure S6: Computed density signals through the ROI for the ultrasound waves of different frequencies and an amplitude of $0.50P^{ext}$ at time $t = t_0$, simulated by employing the momentum-flux-exchanging *setup 2* with $\gamma_{\parallel,\mathrm{ROI}} = 0.0M_{\mathrm{DPD}}/\tau_{\mathrm{DPD}}$.

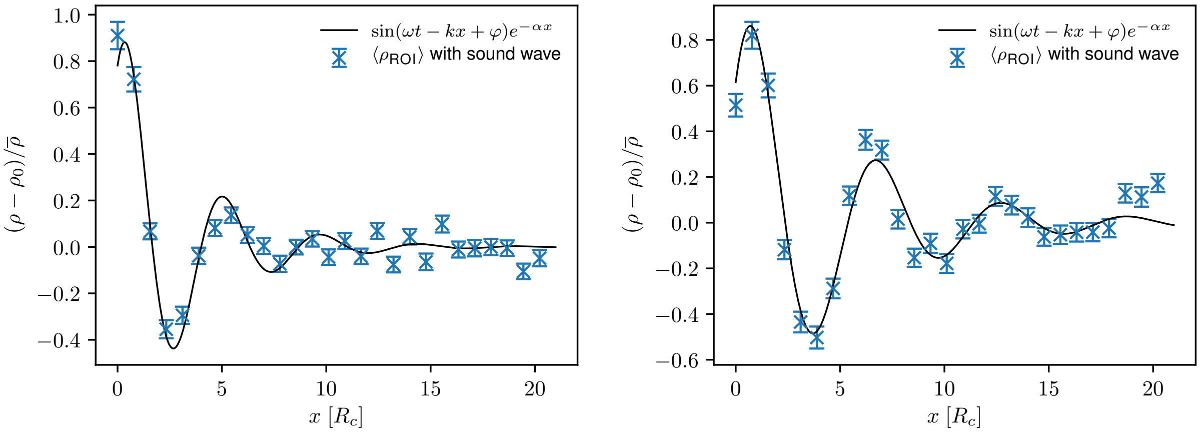

(a) $\nu = 2.76\tau_{\mathrm{DPD}}^{-1}$ (b) $\nu = 2.15\tau_{\mathrm{DPD}}^{-1}$

Figure S7: Same as in Figure S6 but for $\gamma_{\parallel,\mathrm{ROI}} = 2.0M_{\mathrm{DPD}}/\tau_{\mathrm{DPD}}$.

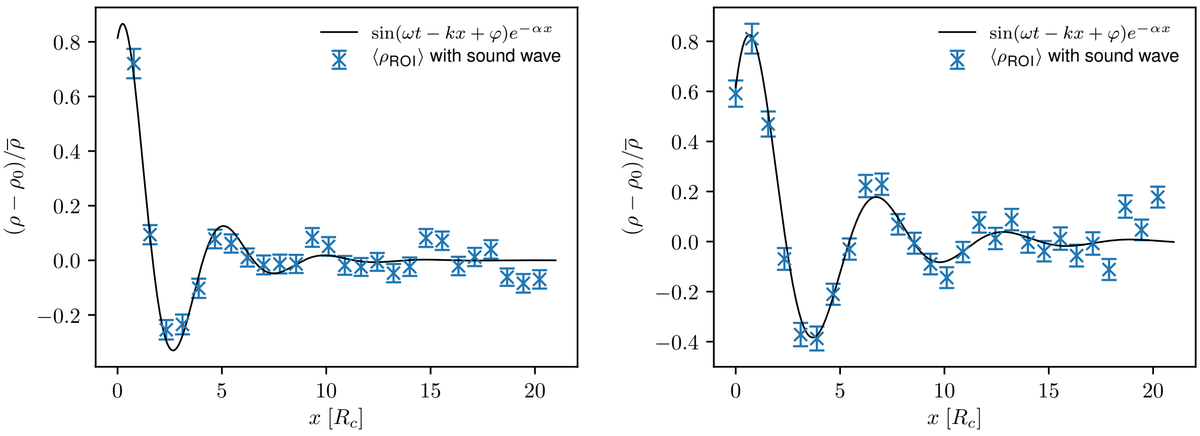

(a) $\nu = 2.76\tau_{\mathrm{DPD}}^{-1}$ (b) $\nu = 2.15\tau_{\mathrm{DPD}}^{-1}$

Figure S8: Same as in Figure S6 but for $\gamma_{\parallel,\mathrm{ROI}} = 4.0M_{\mathrm{DPD}}/\tau_{\mathrm{DPD}}$.

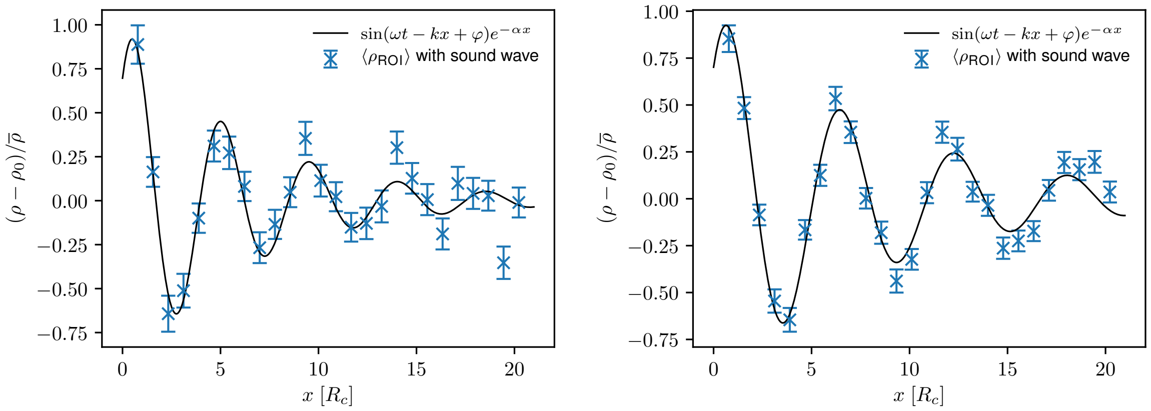

(a) $\nu = 2.76\tau_{\mathrm{DPD}}^{-1}$ (b) $\nu = 2.15\tau_{\mathrm{DPD}}^{-1}$

Figure S9: Same as in Figure S5 for an amplitude of $0.25P^{ext}$ and energy-flux-exchanging *setup 3*.

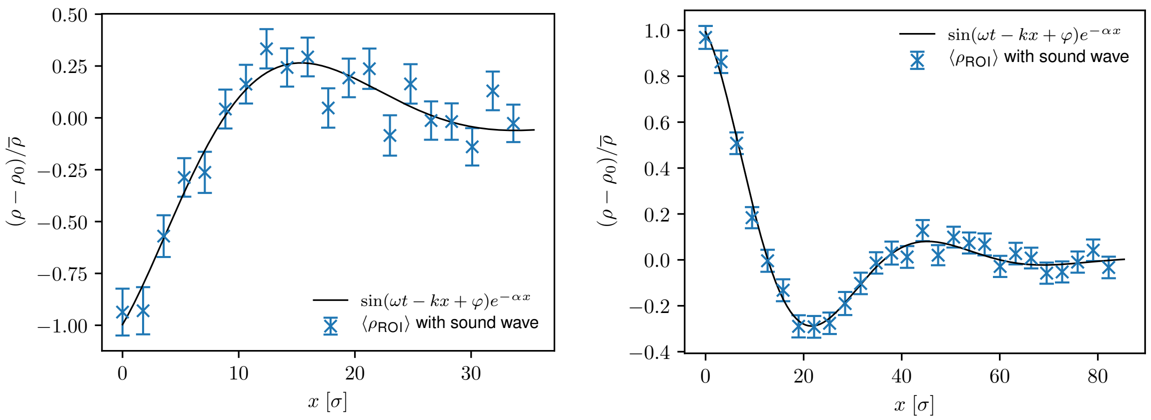

(a) $\nu = 0.31\tau_{\mathrm{MD}}^{-1}$ (b) $\nu = 0.21\tau_{\mathrm{MD}}^{-1}$

Figure S10: Same as in Figure S5 for an amplitude of $2.0P^{ext}$ and AdResS simulation.
